# Ultrasound contrast-enhanced imaging and in vitro antitumor effect of paclitaxel-poly(lactic-co-glycolic acid)-monomethoxypoly (ethylene glycol) nanocapsules with ultrasound-targeted microbubble destruction

**DOI:** 10.3892/mmr.2014.3072

**Published:** 2014-12-10

**Authors:** JING MA, LING XI XING, MING SHEN, FAN LI, MING JIE ZHU, LI FANG JIN, ZHAOJUN LI, FENG GAO, YIJIN SU, YOU RONG DUAN, LIAN FANG DU

**Affiliations:** 1 Department of Ultrasound, Shanghai First People’s Hospital Affiliated to Shanghai Jiao tong University School of Medicine, Shanghai 200080, P.R. China; 2 Department of Cardiovascular Ultrasound, Shanghai East Hospital Affiliated to Tong ji University, Shanghai 200120, P.R. China; 3 Cancer Institute, Renji Hospital Affiliated to Shanghai Jiao tong University School of Medicine, Shanghai 200032, P.R. China

**Keywords:** nanocapsules, paclitaxel-poly(lactic-co-glycolic acid)-monomethoxy poly(ethylene glycol), ultrasound contrast-enhanced imaging, ultrasound-targeted microbubble destruction, antitumor

## Abstract

A combination of diagnostic and therapeutic ultrasound (US) techniques may be able to provide the basis of specific therapeutic protocols, particularly for the treatment of tumors. Nanotechnology may aid the progression towards the use of US for tumor diagnosis and targeted therapy. The current study investigated *in vivo* and *in vitro* US contrast imaging using nanocapsules (NCs), and also US and US-targeted microbubble destruction (UTMD) therapy using drug-loaded NCs for pancreatic cancer *in vitro*. In the current study, the NCs were made from the polymer nanomaterial poly(lactic-co-glycolic acid)-monomethoxypoly(ethylene glycol) (PLGA-mPEG), encapsulated with paclitaxel (PTX), to create PTX-PLGA-mPEG NCs. The PTX-PLGA-mPEG NCs were used as a US contrast agent (UCA), which produced satisfactory US contrast-enhanced images *in vitro* and *in vivo* of the rabbit kidneys, with good contrast compared with lesions in the peripheral regions. However, clear contrast-enhanced images were not obtained using PTX-PLGA-mPEG NCs as a UCA, when imaging the superficial pancreatic tumors of nude mice *in vivo.* Subsequently, fluorescence and flow cytometry were used to measure the NC uptake rate of pancreatic tumor cells under various US or UTMD conditions. An MTT assay was used to evaluate the efficiency of PTX and PTX-PLGA-mPEG NCs in killing tumor cells following 24 or 48 h of US or UTMD therapy, compared with controls. The specific US or UTMD conditions had been previously demonstrated to be optimal through repeated testing, to determine the conditions by which cells were not impaired and the efficiency of uptake of nanoparticles was highest. The current study demonstrated high cellular uptake rates of PLGA-mPEG NCs and high tumor cell mortality with PTX-PLGA-mPEG NCs under US or UTMD optimal conditions. It was concluded that the use of NCs in US-mediated imaging and antitumor therapy may provide a novel application for US.

## Introduction

Ultrasound (US) imaging has been a primary choice for the diagnosis and evaluation of tumors, as it is safe, a real-time measurement, low-cost and portable. The wide use of US contrast agents (UCAs), which may enhance the comparison between lesions and surrounding tissue, has greatly improved the resolution and sensitivity of clinical US imaging (1). Micro-sized UCAs cannot pass through the vascular endothelium, so are consequently regarded as blood pool tracers, nano-scale UCAs however, are able to pass through gaps in the vascular endothelium into tumor mesenchyma. As a result, much cancer therapy research has focused on drug-loaded nanoparticles (NPs). A number of antitumor drugs, including cisplatin (2), mitoxantrone (3), DNA and siRNA (4) have been successfully embedded into NPs, which may provide protection from direct degradation by nucleases *in vivo*. In addition, NPs may facilitate drug uptake into target cells or tissues via an endocytic pathway (5). Previously, studies have undertaken investigations to assess the treatment of pancreatic cancer by drug-loaded NPs (6). However, the results obtained suggested that the efficiency of drug-loaded NP uptake into the tumor tissues remained low. US-targeted microbubble destruction (UTMD) may be an effective method to facilitate NP uptake into various types of tumor tissue *in vivo*, due to an alteration in the permeability of the vasculature and cell membrane (7–10). The aims of the current study were as follows: (i) To evaluate the use of US contrast imaging with a novel NC *in vivo* and *in vitro*; and (ii) to establish the effectiveness of US and UTMD in promoting the uptake of this paclitaxel-loaded NC (PTX-NC) *in vitro* into pancreatic cancer cells to induce cytotoxicity.

## Materials and methods

### Materials

Poly(lactic-co-glycolic acid)-monomethoxypoly(ethylene glycol) (PLGA-mPEG) (LA:GA, 8:2; PEG2000, 10%) was obtained from Shanghai Cancer Institute (Shanghai, China). Pluronic F68 was obtained from BASF Co., Ltd. (Shanghai, China). PTX and dichloromethane were obtained from Tianjin Kaitong Chemical Reagent Co., Ltd. (Tianjin, China). Rhodamine (Rh) and fluorescein isothiocyanate (FITC) were obtained from Beijing Biosea Biotechnology Co., Ltd. (Beijing, China). SonoVue (Bracco, Milan, Italy) is a lipid-coated UCA with sulfur hexafluoride gas microbubbles (MBs), composed of ~2×10^8^ MBs/ml, and an average diameter of 2.5–6.0 μm. A total of 10 female New Zealand white rabbits (12 weeks old; average weight, 3128.54±102.32 g) and 30 nude female BALB/c mice (4 weeks old; average weight, 13.87±1.92 g) were supplied by the First People’s Hospital Affiliated to Shanghai Jiao tong University (Shanghai, China), and all animal procedures were performed in accordance with the research protocol approved by the Animal Care and Use Committee of the hospital.

### Preparation and physicochemical characteristics of the PTX-PLGA-mPEG NCs

The PTX-mPEG-PLGA NCs were prepared using the double-emulsion method (11). PTX solution (1.25 mg in 1.25 ml methanol solution; Shanghai Baoman Biotechnology Co., Ltd., Shanghai, China) was emulsified in the PLGA-mPEG solution (25 mg in 1 ml dichloromethane solution) by sonication (200 W, 5 sec-2 sec-15) using an ultrasonic cell disrupter (JY92-ZD, Ningbo Xinzhi Biotechnology Co., Ltd., Ningbo, China). Subsequently, 10 ml F68 aqueous solution (1 mg/ml) was rapidly added to the first emulsion and sonicated (200 W, 5 sec-2 sec-15). The resultant emulsions were stirred to evaporate the dichloromethane and were then lyophilized (EPSILON 2–6D; Martin Christ, Osterode am Harz, Germany). The NC sizes were measured using an H-7000 Transmission Electron Microscope (Hitachi Ltd., Tokyo, Japan). The size distribution and ζ potential of the NPs in aqueous solution was determined using a Nicomp-380ZLS ζ potential analyzer, from Particle Sizing Systems, Inc. (Port Richey, FL, USA). The drug-loading rate was calculated as the ratio of the amount of PTX encapsulated in NCs to the total amount of NCs (3 mg) initially used. The detection of drug-loading rate was completed by the Shanghai Cancer Institute (Shanghai, China).

### Cell culture

Human pancreatic cancer cells (Aspc-1; Shanghai Tumor Institute, Shanghai, China) were incubated in Dulbecco’s modified Eagle’s medium (Gibco Life Technologies, Grand Island, NY, USA), then were maintained in 10% fetal bovine serum (Sigma, St. Louis, MO, USA), penicillin and streptomycin (100 μg/ml; Shanghai Baoman Biotechnology Co, Ltd.) at 37°C in humidified conditions with 5% CO_2_. Subsequently, the pancreatic cancer cells were seeded into 6- and 96-well plates according to the different experimental conditions.

### US contrast-enhanced imaging analysis of PTX-PLGA-mPEG NCs (nano-UCA) in vitro

The Philips iE33 xMATRIX US system (Philips Healthcare, Andover, MA, USA) was used with an L11-3 probe. A total of 5 ml degassed water was poured into one 5-ml soft tube. Nano-UCA powder (60 mg) was placed into another 5-ml soft tube and topped up with degassed water. The lids of the two tubes were then sealed tightly and the tube containing the nano-UCA solution was vibrated in order to ensure that the powder was completely dissolved prior to imaging. The outer surfaces of the soft tubes were covered with nano-UCA to prevent any air getting between the tubes and the transducer. US is attenuation in air, therefore, ultrasonic medicinal coupling gel was smeared between the tube and the transducer. US contrast images of the nano-UCA solution (12 mg/ml) were immediately collected and recorded.

### US contrast-enhanced imaging analysis of PTX-PLGA-mPEG NCs (nano-UCA) in vivo

The Acuson Sequoia 512 US system (Siemens, Erlangen, Germany) and LOGIQ E9 (GE Healthcare, Wauwatosa, WI, USA) were used with the 15L8W-S and ML6-14 probes, respectively. Degassed water (2 ml) and nano-UCA solution (2 ml; 12 mg/ml) were injected into the ear veins of the rabbits, while US contrast images of the right kidney were observed in real-time and recorded. Similarly, 1 ml degassed water, 1 ml nano-UCA (12 mg/ml) solution and 1 ml SonoVue suspension were injected into the tail veins of the mice, while US contrast images of superficial pancreatic tumors in nude mice were also observed in real-time and recorded. Rabbits were euthanized via injection of 1,200 mg/kg nembutal injected into the ear vein and mice were sacrificed by decapitation.

### Detection of cellular uptake of PLGA-mPEG NCs by fluorescence microscopy

Aspc-1 pancreatic cancer cells (3×10^5^/well) were cultured in two 6-well plates and incubated for 24 h. A therapeutic US machine (Physiomed Elektromedizin AG, Schnaittach, Germany) was used at a frequency of 1 MHz, with the optimal US conditions (power, 1 W/cm^2^; exposure time, 60 sec; duty cycle, 20%; SonoVue volume ratio, 1:5). The cells were divided into three groups as follows: The phosphate-buffered saline (PBS), Rh and Rh-PLGA-mPEG NC group. Each group was exposed to three environmental conditions: i) Control; ii) US; and iii) UTMD. The volume of solution in each well was 1 ml, with an equal volume of Rh. Each group was evaluated and imaged using fluorescence microscopy following 5 h of the respective treatments.

### Detection of cellular uptake of PLGA-mPEG NCs by flow cytometry

The Aspc-1 cells were divided into four groups: The controls (Aspc-1 cells only), the FITC-PLGA-mPEG NCs, FITC-PLGA-mPEG NCs + US and FITC-PLGA-mPEG NCs + UTMD groups. The cells of each group were seeded at a density of ~5×10^5^–1×10^6^ cells/plate. The groups were exposed to the same optimal US conditions (power, 1 W/cm^2^; exposure time, 60 sec; duty cycle, 20%; SonoVue volume ratio, 1:5). The cells in each plate were washed twice with PBS subsequent to administration, and promptly harvested by trypsinization (Aladdin Industrial, Inc., Nashville, TN, USA). Subsequently, the cells were suspended in 1 ml PBS. In the FITC-PLGA-mPEG NCs + UTMD group, 200 μl MB solution (SonoVue) was injected into the 1 ml PBS solution for each plate. Samples were analyzed 5 h subsequent to administration of NC, using a flow cytometer (EPICS XL/XL-MCL; Beckman Coulter, Miami, FL, USA).

### Cellular cytotoxicity test

The cellular cytotoxicity of the NCs was determined by MTT assay. Briefly, the Aspc-1 pancreatic cancer cells (1×10^5^/well) were cultured in 96-well plates and incubated for 24 h. PBS solution; SonoVue dissolved in sterile saline solution (Nanjing Bianzhen Biotechnology Co., Ltd., Nanjing, China); blank PLGA-mPEG NCs; blank PLGA-mPEG NCs + US; blank PLGA-mPEG NCs + UTMD; PTX-PLGA-mPEG NCs; PTX-PLGA-mPEG NCs + US; PTX-PLGA-mPEG NCs + UTMD; and PTX were added to the cells at different concentrations, and incubated for 24 and 48 h at 37°C. The optimal US conditions (power, 1 W/cm^2^; exposure time, 60 sec; duty cycle, 20%; SonoVue volume ratio, 1:5) were used. Subsequently, 0.2 ml MTT (0.5 mg/ml) was added to the culture and incubated for an additional 4 h at 37°C. The culture medium was then removed from the wells and replaced with 0.2 ml dimethyl sulfoxide. Following agitation of the 96-well plates for 15–20 min on a swing bed, the absorbance was measured at a wavelength of 450 nm using a Model 680 Microplate Reader from Bio-Rad Laboratories (Hercules, CA, USA).

### Statistical analysis

Student’s t-test was utilized to identify the significance of differences between the experimental and control groups using SPSS, version 17.0 (SPSS, Inc., Chicago, IL, USA). P<0.05 was considered to indicate a statistically significant difference. All experiments were conducted in triplicate.

## Results

### Characterization and drug-loading rate of PTX-PLGA-mPEG NCs

**Figure 1:**
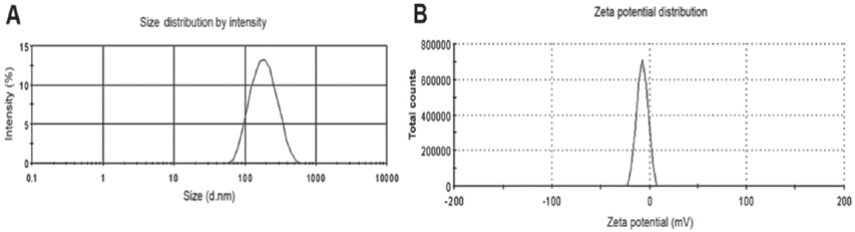
(A) Size distribution and (B) ζ potential of paclitaxel-poly(lactic-co-glycolic acid)-monomethoxy poly(ethylene glycol) nanocapsules.

**Figure 2:**
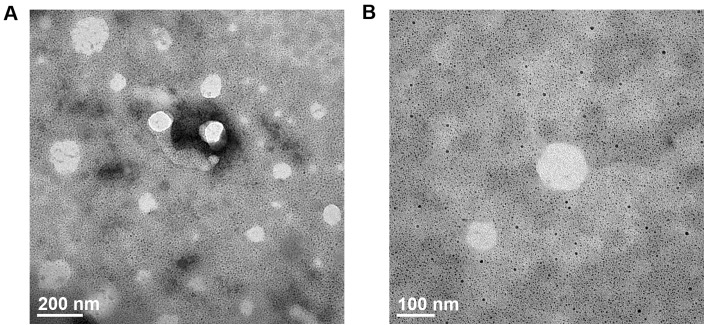
Transmission electron microscope images of paclitaxel-poly(lactic-co-glycolic acid)-monomethoxy poly(ethylene glycol) nanocapsules. Magnification (A) ×10,000 and (B) ×30,000.

The results from the particle size and ζ potential analyzer demonstrated that the PTX-PLGA-mPEG NC sizes were between 85.75 and 632.43 nm; the average size was 276.38 nm and the ζ potential was −6.94 mV (Fig. 1). Observed by transmission electron microscopy (TEM; Olympus IX53; Olympus, Tokyo, Japan), the morphology of the PTX-PLGA-mPEG NCs were spherical with advanced dispersion and no aggregation. The inside of the PTX-NCs exhibited hollow honeycomb-like holes as observed in the TEM images shown in Fig. 2. The drug-loading rate of PTX-NCs was calculated to be 1.6%.

### US contrast-enhanced imaging of PTX-PLGA-mPEG NCs

**Figure 3:**
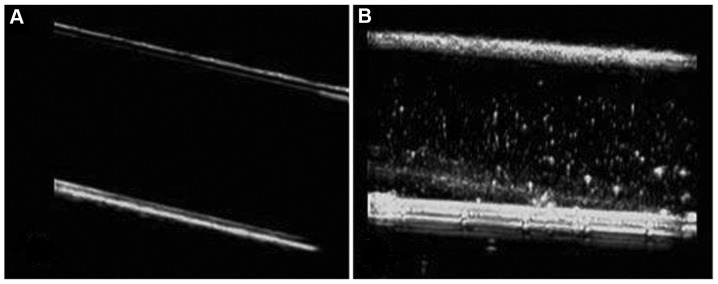
Ultrasound contrast-enhanced images *in vitro*. (A) Tube filled with degassed water; (B) tube filled with paclitaxel-poly(lactic-co-glycolic acid)-monomethoxy poly(ethylene glycol) nanocapsule solution.

**Figure 4:**
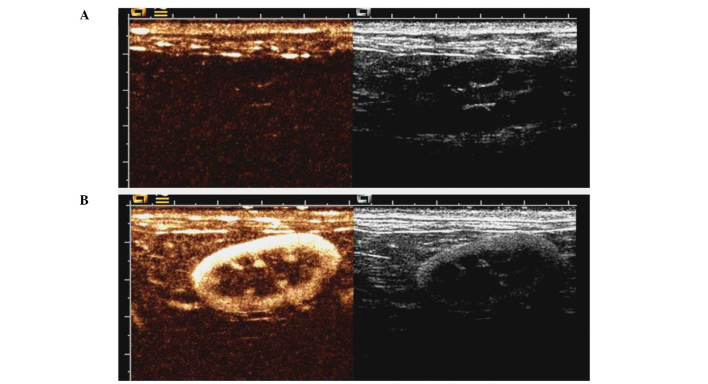
Representative *in vivo* ultrasound contrast-enhanced images in the rabbit right kidney. (A) Pre- and (B) post-administration of paclitaxel-poly(lactic-co-glycolic acid)-monomethoxy poly(ethylene glycol) nanocapsule solution. Left, contrast mode; right, conventional B-mode.

**Figure 5:**
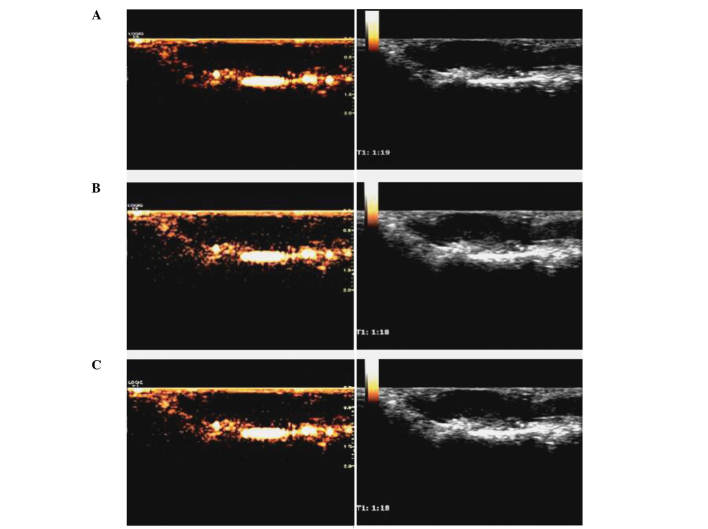
Representative i*n vivo* ultrasound contrast-enhanced images of pancreatic superficial tumors in nude mice. (A) Pre- and (B) post-administration of paclitaxel-poly(lactic-co-glycolic acid)-monomethoxy poly(ethylene glycol) nanocapsule solution; and (C) post-administration of SonoVue. Left, contrast mode; right, conventional B-mode.

Observed from US contrast-enhanced images, a tube filled with PTX-PLGA-mPEG NC UCA displayed a strong dotted-echo, whereas a tube filled with degassed water was observed as black (Fig. 3). Imaging of the rabbit right kidney *in vivo* following PTX-PLGA-mPEG NC UCA administration resulted in excellent contrast-enhanced images, whilst unclear images were observed pre-administration (Fig. 4). However, the contrast-enhanced images of superficial pancreatic tumors in nude mice following administration of PTX-PLGA-mPEG NC UCA and SonoVue suspension were unclear, similar to the level of clarity prior to administration (Fig. 5).

### Detection of cellular uptake of NCs by fluorescence microscopy

**Figure 6:**
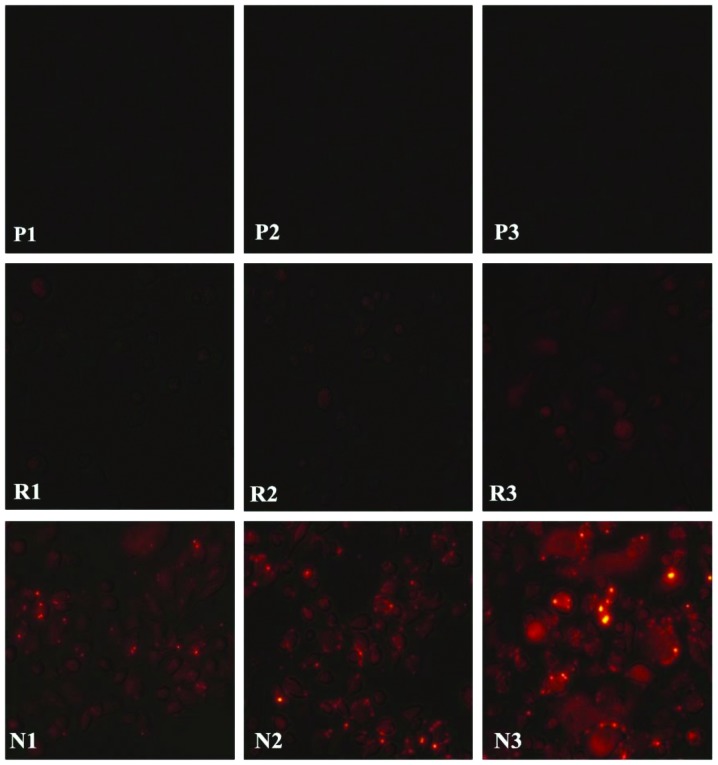
Intracellular uptake of Rh-PLGA-mPEG NCs following 5 h of different conditions. Representative images displaying the fluorescence of the 9 groups, observed under the fluorescence microscope. P, PBS group; R, Rh group; N, Rh-PLGA-mPEG NCs group. 1, no US; 2, with US; 3, with UTMD. Rh-PLGA-mPEG NCs, rhodamine-poly(lactic-co-glycolic acid)-monomethoxy poly(ethylene glycol) nanocapsules; PBS, phosphate-buffered saline; Rh, rhodamine; US, ultrasound; UTMD, US-targeted microbubble destruction.

As presented in Fig. 6, greater fluorescence was observed in Aspc-1 cells 5 h subsequent administration of the Rh-PLGA-mPEG NC solution (panels N1–3), compared with the control groups [PBS (panels P1–3) and Rh only (panels R1–3)]. Furthermore, greater fluorescence was observed in Aspc-1 cells following administration of the Rh-PLGA-mPEG NC solution under the condition of UTMD (panel N3) compared with US (panel N2). No marked fluorescence was observed in the control groups (PBS and Rh only groups) under any of the conditions (panels P1–3 and R1–3).

### Quantification of cellular uptake of NCs by flow cytometry

**Figure 7:**
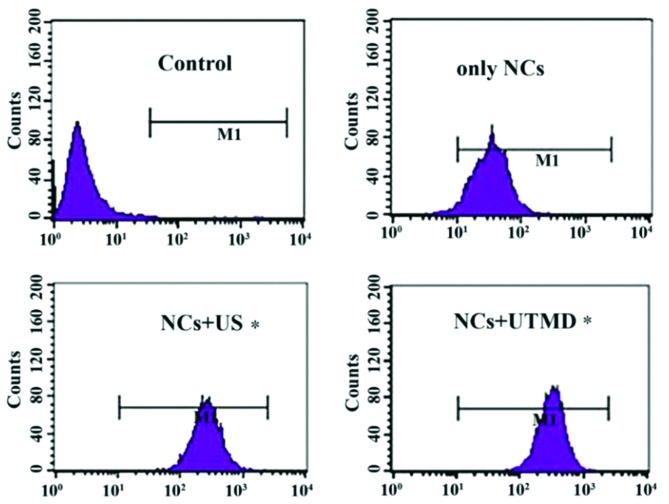
Flow cytometry results indicating the cell fluorescence of Aspc-1 cells in each group (controls, 0.05%; NCs only, 23.42%; NCs + US, 77.35%; and NCs + UTMD, 81.47%). P<0.05 in the NC + US and NC + UTMD, compared with the only NC group. NCs, nanocapsules; US, ultrasound, UTMD, US-targeted microbubble destruction; M1, gate.

In the NCs + US and NCs + UTMD groups, the intracellular uptake rates were significantly greater than in the group with NCs alone (P<0.05). The NC uptake efficiency was not significantly higher in the NCs + UTMD group compared with that of the NCs + US group (Fig. 7)

### MTT assay

**Figure 8:**
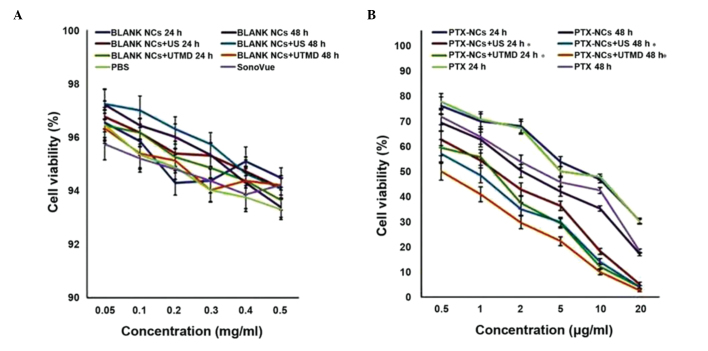
MTT assay. The cell viabilities of Aspc-1 pancreatic cancer cells 24 or 48 h subsequent to different treatments. (A) Blank nanocapsules; (B) paclitaxel-poly(lactic-co-glycolic acid)-monomethoxypoly (ethylene glycol) nanocapsules.

The cell viabilities of Aspc-1 pancreatic cancer cells following administration of PBS, SonoVue, blank NCs, blank NCs + US, or blank NCs + UTMD for 24 or 48 h were all between 92 and 96% (Fig. 8A). No significant differences in cell viability were identified between these groups (P>0.05). In the PTX-NC groups, it was identified that the NCs did not significantly affect cell viability in the absence of US or UTMD compared with PTX treatment alone (P>0.05). However, in the US or UTMD groups, following incubation for 24 or 48 h, the PTX-NC cytotoxicities towards the cells were indicated to be significantly higher than those that underwent treatment with PTX alone (P<0.05). Furthermore, subsequent to incubation for 24 or 48 h, the cell viabilities of the pancreatic cancer cells with PTX-NCs mediated by UTMD were significantly lower than those mediated by US (P<0.05) (Fig. 8B).

## Discussion

Conventional UCAs, such as the lipid-shelled SonoVue filled with sulfur hexafluoride gas, have been widely used in clinical practice. These types of UCAs have the ability to produce useful contrast-enhanced images, but cannot act therapeutically. It has been demonstrated that drugs and genes are able to adhere to the surface of the UCAs, or be encapsulated into them to be used therapeutically (12). The current study designed a novel nano-UCA that can be used not only in contrast-enhanced imaging, but also to deliver drugs into tumor cells.

This novel nano-UCA was made using PLGA-mPEG, a commonly used biodegradable polyester material, which has been approved to be nontoxic and harmless by the US Food and Drug Administration (13). An mPEG molecule can prolong the body circulation time of drugs and increase time at the tumor tissue by reducing their recognition by the reticuloendothelial system, thus an mPEG molecule may be beneficial in the treatment of tumors *in vivo*. The double-emulsion method was used for the preparation of PTX-PLGA-mPEG NCs, which were observed by TEM to be spherical in shape, with a diameter ranging between 85.75 and 632.43 nm. During the course of synthesis, the encapsulated water in the inner aqueous phase of the NCs was sublimated by lyophilization. This resulted in small hollow holes with a honeycomb structure, providing a basis for the US-responsive properties. The properties of PTX-PLGA-mPEG NCs following lyophilization were stable; this type of nano-UCA powder may be kept for a long time at a temperature of −20°C without alteration to its appearance. Furthermore, the morphology observed by TEM following resolution revealed good dispersion and no aggregation.

It was also observed that PTX-PLGA-mPEG NCs yielded effective contrast-enhanced images *in vitro* and in rabbit right kidney *in vivo*. However, contrast images in superficial pancreatic cancer tumors in nude mice were not satisfactory with administration of PTX-PLGA-mPEG NCs or with SonoVue solution. A potential explanation may be as follows: In general, the vessels of the tumor were divided into two sections; one section consisted of the original vessels, the endothelial gaps of which were <100 nm. The other section was composed of newly formed vessels, the endothelial structure of which was not intact and did not contain smooth muscle. Due to this, the endothelial gaps in these newly formed blood vessels were larger; between 380–780 nm (14). As a result of this, a greater nano-UCA influx into the tumor tissues may have occurred, compared with micro-UCAs. The pancreatic tumor tissues of nude mice and humans presented specific pathological and anatomical similarities, with dense, poorly vascularized connective tissues immersed in a large volume of fibrous tissues and lymphocytes (15). Hence, to a certain extent, nano-UCAs may produce improved contrast-enhanced images in rabbit kidney and maintain a sufficient blood supply, compared with those in superficial tumors of nude mouse pancreas. Additionally, the hollow holes in lyophilized PTX-PLGA-mPEG NCs may be so small that they lead to strong ultrasonic reflection, and a higher concentration of the nano-UCA solution would be required for US contrast imaging in pancreatic tumors.

The present study demonstrated a greater Rh-PLGA-mPEG NC uptake (red fluorescent signals in Fig. 6) in Aspc-1 cells following administration of Rh-PLGA-mPEG NCs mediated by US or UTMD (N2 and 3), when comparing intracellular uptake ratios among the PBS, Rh alone and Rh-PLGA-mPEG groups in US (N1), US (N1) and UTMD (N3) conditions. Furthermore, stronger red fluorescent signals in Aspc-1 cells were observed following administration of the Rh-PLGA-mPEG NCs with UTMD (N3) compared with US (N2). Similar results were observed when measuring NC cellular uptake with flow cytometry. The cellular uptake efficiencies of the PLGA-mPEG NC groups with US and UTMD were higher than the uptake of the PLGA-mPEG NCs alone. The PLGA-mPEG NC cellular uptake efficiency in the PLGA-mPEG NCs + UTMD group was not significantly higher than in the PLGA-mPEG NCs + US group. These results indicate that US and UTMD are effective driving forces that may be favorable methods to increase PLGA-mPEG NC uptake to Aspc-1 pancreatic cancer cells.

Possible mechanisms for promoting NP uptake into cells have been studied, but the most efficient mechanism remains unclear: Micro-circumflex or micro-fluid generation by UTMD punching transient holes in the cell membrane surface (16); an increase in reactive oxygen species in cells; cell membrane transport abilities becoming activated; and an increase in cell membrane temperature during US (17–21) are a number of possibilities. Furthermore, a previous study proposed a novel mechanism, that UTMD may stimulate cellular clathrin-dependent endocytosis (22).

In the MTT assay, the cell viabilities of Aspc-1 pancreatic cancer cells following administration of PBS, SonoVue or blank PLGA-mPEG NCs with or without US or UTMD for 24 or 48 h were all above 90%. This demonstrated that the cellular cytotoxicity of the blank PLGA-mPEG NCs at each concentration tested was negligible, and also that the optimal US and UTMD conditions had almost no effect on these Aspc-1 cells *in vitro* without the PTX-PLGA-mPEG NCs. However, when combined with PTX-PLGA-mPEG NCs, the cytotoxicity was greater following US and UTMD than with no US. In addition, due to more powerful sonoporation, UTMD elicited an increased NC uptake into the Aspc-1 cells compared with US. Thus, US and UTMD were demonstrated to be powerful physical techniques, which may safely and efficiently deliver PTX-PLGA-mPEG NCs into Aspc-1 cells *in vitro*, consequently producing an antitumorigenic effect.

In conclusion, a novel PTX-PLGA-mPEG NC technique, which combined US contrast imaging and antitumor therapy, was successfully designed and prepared. UTMD, a promising physical targeting vehicle, may facilitate improved PTX-PLGA-mPEG NC uptake into Aspc-1 pancreatic cancer cells and enhanced antitumorigenic action *in vitro*. Thus, the combination of nanotechnology and US may present a novel method for monitoring and treating tumors. Further study is required to continue the investigation of US-specific contrast imaging and antitumor treatment, and future studies should include a variety of tumor types *in vivo*.
